# Accuracy of different diagnostic techniques for *Schistosoma haematobium* to estimate treatment needs in Zimbabwe: Application of a hierarchical Bayesian egg count model

**DOI:** 10.1371/journal.pntd.0008451

**Published:** 2020-08-20

**Authors:** Nicholas Midzi, Oliver Bärenbold, Portia Manangazira, Isaac Phiri, Masceline J. Mutsaka-Makuvaza, Gibson Mhlanga, Jürg Utzinger, Penelope Vounatsou

**Affiliations:** 1 National Institute of Health Research, Ministry of Health and Child Care, Harare, Zimbabwe; 2 Swiss Tropical and Public Health Institute, Basel, Switzerland; 3 Faculty of Science, University of Basel, Basel, Switzerland; 4 National Institute of Health Research, Ministry of Health and Child Care, Harare, Zimbabwe; 5 Department of Medical Microbiology, University of Zimbabwe, Harare, Zimbabwe; Weill Cornell Medical College, UNITED STATES

## Abstract

**Background:**

Treatment needs for *Schistosoma haematobium* are commonly evaluated using urine filtration with detection of parasite eggs under a microscope. A common symptom of *S*. *haematobium* is hematuria, the passing of blood in urine. Hence, the use of hematuria-based diagnostic techniques as a proxy for the assessment of treatment needs has been considered. This study evaluates data from a national survey in Zimbabwe, where three hematuria-based diagnostic techniques, that is microhematuria, macrohematuria, and an anamnestic questionnaire pertaining to self-reported blood in urine, have been included in addition to urine filtration in 280 schools across 70 districts.

**Methodology:**

We developed an egg count model, which evaluates the infection intensity-dependent sensitivity and the specificity of each diagnostic technique without relying on a ‘gold’ standard. Subsequently, we determined prevalence thresholds for each diagnostic technique, equivalent to a 10% urine filtration-based prevalence and compared classification of districts according to treatment strategy based on the different diagnostic methods.

**Principal findings:**

A 10% urine filtration prevalence threshold corresponded to a 17.9% and 13.3% prevalence based on questionnaire and microhematuria, respectively. Both the questionnaire and the microhematuria showed a sensitivity and specificity of more than 85% for estimating treatment needs at the above thresholds. For diagnosis at individual level, the questionnaire showed the highest sensitivity (70.0%) followed by urine filtration (53.8%) and microhematuria (52.2%).

**Conclusions/Significance:**

The high sensitivity and specificity of a simple questionnaire to estimate treatment needs of *S*. *haematobium* suggests that it can be used as a rapid, low-cost method to estimate district prevalence. Our modeling approach can be expanded to include setting-dependent specificity of the technique and should be assessed in relation to other diagnostic methods due to potential cross-reaction with other diseases.

## Author summary

The strategy of the World Health Organization (WHO) to combat schistosomiasis is based on administering praziquantel as preventive chemotherapy to school-age children only, or including adults. For *Schistosoma haematobium* the treatment decision is based on observed prevalence by urine filtration which involves counting eggs under a light microscope. Urine filtration is time intensive and requires trained technicians to operate the microscope. Blood in urine is a common symptom of urogenital schistosomiasis and therefore, the use of screening tools based on the detection of hematuria has been suggested. We analyze three such screening tools, namely reagent strip to detect microhematuria, a questionnaire regarding recent history of blood in urine, and visible inspection of urine and determine their diagnostic accuracy to evaluate treatment need at district level in comparison to urine filtration in a national survey in Zimbabwe. We find that both the questionnaire and reagent strip classify districts with sensitivity and specificity above 85% at the 10% urine filtration thresholds. Wrongly classified districts generally show urine filtration prevalence in the vicinity of the 10% prevalence thresholds. We conclude that there is evidence that a questionnaire can be used as a low cost alternative rapid screening tool for *S*. *haematobium*.

## Introduction

Schistosomiasis is one of the world’s neglected tropical diseases causing morbidity primarily in school-age children but affecting the whole population in rural areas of low- and middle-income countries where there is little or no sanitation and clean water [1–3]. Urogenital schistosomiasis, caused by the bloodfluke *Schistosoma haematobium* is highly endemic in many countries of Africa and the Middle East [2, 3]. Hematuria, dysuria, cystitis, and strictures associated with the bladder and ureter, as well as hydronephrosis and renal failure are sequelae of advanced cases of urogenital schistosomiasis [4]. Carcinoma of the bladder is also a late complication [5, 6].

At the 54th World Health Assembly in 2001, the World Health Organization (WHO) encouraged member states to regularly provide mass drug administration (MDA) using praziquantel to at least 75% of the school-age children at risk of morbidity due to schistosomiasis [7, 8]. Subsequently, a manual for control program managers was published, specifying MDA once every year if the prevalence of schistosomiasis is ≥ 50% by a standard parasitologic approach such as urine filtration or if ≥ 30% by morbidity questionnaires (i.e. anamnestic self-reported hematuria). MDA should be given once every 2 years in moderate risk areas (parasitologic prevalence ≥ 10% but < 50%, or prevalence of hematuria ≥ 10% but < 30% [9]).

To target MDA effectively, rapid assessment procedures are needed for identifying high-risk communities [10]. However, there has not been any provision of alternative cost-effective field applicable tools for the rapid screening of urogenital schistosomiasis with superior sensitivity than the standard urine filtration method. At a time when the global target has been shifted from morbidity control to elimination, strategies aiming at rapid transitioning from the state of schistosomiasis infectiousness, and transmission interruption to elimination phase are needed [11]. These phases would naturally require diagnostic tools with high sensitivity and specificity to cater for the diagnostic error demanded at each stage [12]. Whilst urine filtration is the current ‘gold’ standard for the detection of *S*. *haematobium* eggs with high specificity, this method requires trained microscopist and costly consumables, including special filter holders and filter papers that are not locally manufactured in sub-Saharan Africa. The use of hematuria to determine treatment strategies at national level has only been assessed at small scale, as one among the tools that can be applied for screening communities with high risk of schistosomiasis for morbidity control [10, 13–15]. It is not well known whether hematuria would correctly identify and categorize the different endemic areas in a country into specific risk categories, as parasitologic tests do. As part of a plan of action for the control of schistosomiasis and soil-transmitted helminthiasis in Zimbabwe, a baseline survey that involved mapping of both urogenital and intestinal schistosomiasis was conducted. Data were collected on history of hematuria, as described by Mott et al. (1985) and Lengeler et al. (2002), as well as macrohematuria and microhematuria. Additionally, urine filtration was employed, which is the most widely used approach for the diagnosis of urogenital schistosomiasis [10, 14, 16].

The aim of this study was to assess the accuracy of three diagnostic methods for *S*. *haematobium*, based on the presence of blood in urine for predicting treatment needs. We extended our earlier egg count model, developed for estimating the diagnostic error of the Kato-Katz method for *S*. *mansoni* and estimated the sensitivity of *S*. *haematobium* diagnostic methods taking into account the dependence of the sensitivity on infection intensity, and the correlation of false positives among the hematuria-based diagnostic methods [17]. Previous analyses were either based on latent class methods, neglecting infection intensity and reducing the urine filtration reading to a binary outcome, or summarizing repeated urine filtration results by a composite value, which is treated as a ‘gold’ standard, thus over-estimating sensitivity and under-estimating specificity [18, 19].

## Materials and methods

### Ethics statement

The proposal to conduct the national schistosomiasis and soil-transmitted helminthiasis survey was approved by the national ethics review board and the Medical Research Council of Zimbabwe. The ethical approval registration number for the study is MRCZ/A/1207. The Secretary for Education, Sport, Arts, and Culture also approved the study. Written informed consent was sought from the parents/guardians of study participants. UNICEF delivered parental/guardian informed consent forms addressed to each school by the Secretary for Education, Sport, Arts, and Culture throughout the country in advance to allow school heads sufficient time to liaise with parents/guardians for their consent. On the day of sample collection, only the assenting children whose consent forms were signed by their parents/guardians were enrolled. Participation was voluntary; hence, children could withdraw from the study at any time without further obligation.

### Study design

A school-based cross-sectional survey was conducted in Zimbabwe to estimate the geographic distribution of schistosomiasis and soil-transmitted helminthiasis in the country; first in rural provinces (September and October 2010), then in the metropolitan provinces of Harare, Bulawayo, and Chitungwiza towns (July and August 2011). The survey was done in collaboration between the Ministry of Health and Child Care (MHCC) and the Ministry of Education, Sport Arts and Culture (MESC). There are a total of 73 districts in Zimbabwe, of which 58 are rural ones, seven are districts in Harare including peri-urban areas, six are districts in Bulawayo, and two are districts in Chitungwiza. The survey was conducted in 70 (95.8%) districts. Three districts (Gweru, Kwekwe, and Bindura) were left out due to limited resources.

Ten teams, each consisting of two laboratory technicians, one technical assistant, one district community nurse, one district education officer, the district environmental health officer and a driver, conducted data collection. Half of the laboratory technicians were drawn from the National Institute of Health Research to lead the teams with the overall responsibilities of organizing and managing field data collection. These technicians were also responsible for performing the urine filtration and the Kato-Katz techniques [20]. The other technicians were drawn from the province and were responsible for performing the formol-ether concentration technique as well as for assisting the team leader in executing other duties [21].

The technical assistants helped in processing specimens and cleaning filters, templates, and sieves for re-use. The community nurses were responsible for visual assessment of blood in urine and treatment of study participants. The district environmental officers ensured clean environment in schools and at every stage during sample collection, as well as providing food to children during treatment. The district education officers located the primary schools randomly selected for the national survey, introduced the research team to the school authorities, and enrolled a random sample of school children into the study.

### Study population

School children aged 10-15 years were targeted for the study as they constitute the high-risk age group for schistosomiasis and soil-transmitted helminth infections in the community [9, 22].

### Sample size calculation and selection of participants

The sample size at national level was based on the total enrollment of primary school children, i.e., n = 2,490,568. A sample of 15,818 children was calculated using Epi Info version 6 (Centers for Disease Control and Prevention, Atlanta, United States of America) assuming an average national prevalence of schistosomiasis equal to 37% and an error margin of 0.75% [23]. The number of children per district was considered proportional to these enrolled nationally. Simple random sampling was used to select schools per district, following the lottery method [22]. At each school, 50 children equally distributed by sex were randomly selected using the lottery approach [8]. While school children aged 10–15 years constituted the desired sampling frame, children aged 6–9 years (n = 598) and some aged above 15 years (n = 6) were included in some schools where the number of children aged 10–15 years was below 50 [16].

### Diagnosis and treatment

A single mid-day urine sample was collected in 100 ml screw cap plastic specimen bottles from each participant [24]. For the quantitative diagnosis of *S*. *haematobium* infection, the standard urine filtration method was applied [25]. The technique involves filtration of 10 ml of a thoroughly mixed urine specimen, through a Nytrile filter (12–14 mm diameter; mesh size: 20 *μm*).

In addition, each individual was screened using the following indirect tests: (i) a questionnaire regarding recent history of hematuria; (ii) inspection of the urine specimen for visible blood; and (iii) use of reagent strips to detect hematuria. To determine the presence and severity of microhematuria, all urine specimens were tested for presence of detectable blood using urine reagent strips (Bayer Hemastix; Leverkusen, Germany). The results were recorded semi-quantitatively, as follows: negative, trace hemolyzed, weakly positive (+), moderately positive (++), and highly positive (+++) according to the manufacturer. Using this test, an individual was diagnosed negative if there was no positive reaction of the reagent strip from trace to highly positive and positive if trace, weakly, moderately, and highly positive was detected. The recent history of blood in urine was assessed asking the following question: “Have you seen blood in your urine in the past month?” [10, 14]. The individual was regarded as positive if the answer was yes, and negative if the answer was no to the question. For macrohematuria, the community nurse assessed each urine specimen for visible blood in urine as they normally do at health facilities. If there was no blood detected in urine, the individual was diagnosed as negative and otherwise as positive.

Following submission of stool and urine samples, all participants received 100 ml of orange juice and a piece of bread to eat after which they simultaneously received a single dose of praziquantel (40 mg/kg) and albendazole (400 mg) in tablet form regardless of their infection status since both drugs are considered safe [9].

### Statistical methods

Data collected from the field were coded into binary variables representing positive or negative for the reagent strip, recent history of blood in urine, and macrohematuria. The urine filtration technique records the number of eggs per 10 ml of urine. Mapping of the disease risk and comparisons between diagnostic methods were done at the district level with data from the 70 districts described previously.

We developed a Bayesian hierarchical model to estimate the diagnostic sensitivity and specificity of the four tests. In particular, we extended our earlier work on *S*. *mansoni* and took into account an infection intensity-dependent sensitivity and specificity of macrohematuria (*M*), microhematuria (*m*), and the questionnaire (*Q*) [17, 26]. The results of the four diagnostic methods were assumed to be only dependent on infection intensity when infection was present, while the three hematuria-based methods were considered to be correlated via a latent variable describing blood in urine for uninfected individuals, that is

$$\begin{array}{c} \begin{array}{cc} P(Y_{i}^{F},Y_{i}^{m},Y_{i}^{M},Y_{i}^{Q}) & =P\left(Y_{i}^{F}|D_{i}=1\right)P\left(Y_{i}^{m}|D_{i}=1\right)P\left(Y_{i}^{M}|D_{i}=1\right)P\left(Y_{i}^{Q}|D_{i}=1\right)\pi_{d}\\ & +P\left(Y_{i}^{F}|D_{i}=0\right)P\left(Y_{i}^{m},Y_{i}^{M},Y_{i}^{Q}|D_{i}=0\right)\left(1-\pi_{d}\right) \end{array} \end{array}$$ (1)

where $Y_{i}^{F}$ is the urine filtration egg count for individual *i*, $Y_{i}^{m}$ is the binary result of the microhematuria test including traces, $Y_{i}^{M}$ is the binary result of the macrohematuria test, and $Y_{i}^{Q}$ indicates the binary result of the questionnaire. *D*_*i*_ is the disease status, taking 1 for infected and 0 for uninfected individuals and *π*_*d*_ is the true infection prevalence of district *d* = 1, 2, …, 70. Urine filtration measurements were modeled by a negative binomial count distribution, that is $P\left(Y_{i}^{F}|D_{i}=1\right)\equiv NB\left(\lambda_{i},k\right)$ and infection intensities, λ_*i*_ were considered to be gamma distributed in the population of infected individuals, λ_*i*_ ∼ *Gamma*(*μ*_*d*_ *α*, *α*), with *μ*_*d*_ describing the mean infection intensity among infected individual in district *d* and *α* specifying the aggregation of infection intensities in the population.

Microhematuria, macrohematuria, and the questionnaire data were modeled by a Bernoulli distribution with the sensitivity depending on the infection intensity of an infected individual, that is,

$$\begin{array}{c} P\left(Y_{i}^{z}|D_{i}\right)=\{\begin{array}{cc} Be(s_{i}^{z})\; & \text{,}\;\text{if}\;D_{i}=1\\ Be(1-c_{i}^{z})\; & \text{,}\;\text{if}\;D_{i}=0 \end{array} \end{array}$$ (2)

The specificity parameter $c_{i}^{z}$ for the *z* diagnostic method was defined as $c_{i}^{z}=c^{z}b_{i}$, where *b*_*i*_ reflects the correlation between false-positives from blood detecting methods. The mean of the $c_{i}^{z}$ was calculated separately for girls aged 12 years and older from the rest of the subjects to investigate influence of menstruation on the probability of false positives. The egg intensity-dependent sensitivity $s_{i}^{z}$ of *z* diagnostic method was described by $\mathrm{logit}\left(s_{i}^{z}a_{3}^{z}\right)=a_{0}^{z}+a_{1}^{z}\lambda_{i}^{\log(a_{2}^{z}+1)}$, where the infection intensity λ_*i*_ corresponds to the number of eggs in 10 ml of urine for individual *i*. $a_{0}^{z}$ determines the sensitivity in the limit of very low infection intensity; $a_{1}^{z}$ quantifies the dependence of sensitivity on infection intensity; $a_{2}^{z}$ determines the shape of the curve; and $a_{3}^{z}$ the sensitivity in the limit of very high infections. We assumed that the sensitivity of the alternative diagnostic methods for an individual depends on infection intensity only and not on other individual variables like age and sex.

To complete Bayesian model formulation, we chose a uniform prior *π*_*d*_ ∼ *U*(0, 1), a gamma distribution with mean 40 and standard deviation (SD) 40 for the mean infection intensity *μ*_*d*_, a normal distribution with mean 0.1 and SD 0.1 for the population variation *α*_*d*_, a normal distribution with mean -1 and SD 1.5 for $a_{0}^{z}$, a gamma distribution with shape and scale parameters 5 and 30 respectively, for $a_{1}^{z}$, a normal distribution with mean 0.5 and SD 0.5 for $a_{2}^{z}$, and a beta distribution with parameters 10 and 1 for $a_{3}^{z}$ to ensure a non-informative distribution of sensitivity curves in the relevant range of infection intensities. Markov chain Monte Carlo (MCMC) simulations were run for 1,000 iterations with 50 chains in Stan version 2.16.2 (Stan Development Team; mc-stan.org) [27]. Convergence was assessed using the Gelman-Rubin diagnostics [28].

## Results

### Demographics

A total of 13,195 school children drawn from 280 schools was included in the study. Of these, 12,656 (95.9%) had all four diagnostic methods performed and results recorded. The mean age was 11.2 years and 95.6% of the children were between the age of 10 and 15 years.

### Country-level prevalence and diagnostic accuracy

Table 1 shows observed results of the diagnostic tests in the study population. The prevalence measured by single urine filtration was 17.6%, by microhematuria 20.8%, by macrohematuria 4.3% and by questionnaire 28.5%, while the cumulative prevalence was 37.6%. The arithmetic mean egg count among those positive by urine filtration was 76.6 eggs/10 ml of urine, and in the full population was 13.5 eggs/10 ml of urine.

**Table 1:** Summary of prevalence and infection intensity from a national survey for schistosomiasis and soil-transmitted helminthiasis conducted in Zimbabwe in 2010/2011 in all provinces except Gweru, Kwekwe, and Bindura.

	Estimate	95% CI ^1^
Urine filtration prevalence (%)	17.6	16.9–18.2
Microhematuria prevalence (%)	20.8	20.1–21.5
Macrohematuria prevalence (%)	4.3	3.9–4.6
Questionnaire prevalence (%)	28.5	27.7–29.3
Cumulative prevalence (%)	37.6	36.7–38.4
Mean infection intensity among those positive by filtration (eggs/10 ml of urine)	76.6	69.3–84.4
Population mean infection intensity (eggs/10 ml of urine)	13.5	12.2–15.0

^1^ Confidence intervals (CI) were determined by bootstrap

Table 2 shows model-based estimates of the diagnostic errors and the ‘true’ prevalence, and mean egg count of all individuals and of the positively tested ones. The overall ‘true’ prevalence was estimated to be 35.3%, which leads to an overall sample sensitivity of 53.8% for urine filtration, 52.2% for microhematuria, 11.5% for macrohematuria, and 70.0% for the questionnaire. The specificity was estimated to be 95.6% for microhematuria, 99.3% for macrohematuria, and 93.7% for the questionnaire. Specificity of the three hematuria-based diagnostic methods did not differ in the 12- to 14- year-old female population. The mean infection intensity of an infected individual was estimated at 45.4 eggs/10 ml of urine and the mean infection intensity of all individuals at 15.4 eggs/10 ml of urine.

**Table 2:** Model-based estimates of the prevalence, diagnostic errors, and mean infection intensity (posterior mean and 95% Bayesian credible intervals, BCI).

	Estimate	95% BCI
‘True’ prevalence (*π*) (%)	35.3	33.7–36.9
Urine filtration sensitivity (%)	53.8	51.3–56.2
Microhematuria sensitivity (%)	52.2	49.7–54.7
Macrohematuria sensitivity (%)	11.5	10.4–12.6
Questionnaire sensitivity (%)	70.0	67.7–72.4
Microhematuria specificity (%)	95.6	95.0–96.2
Macrohematuria specificity (%)	99.3	99.1–99.5
Questionnaire specificity (%)	93.7	92.6–94.7
‘True’ infected mean (*μ*) (%)	45.4	42.2–48.9
‘True’ full mean (*μπ*) (%)	15.4	14.3–16.4

### District-level prevalence

**Fig 1:**
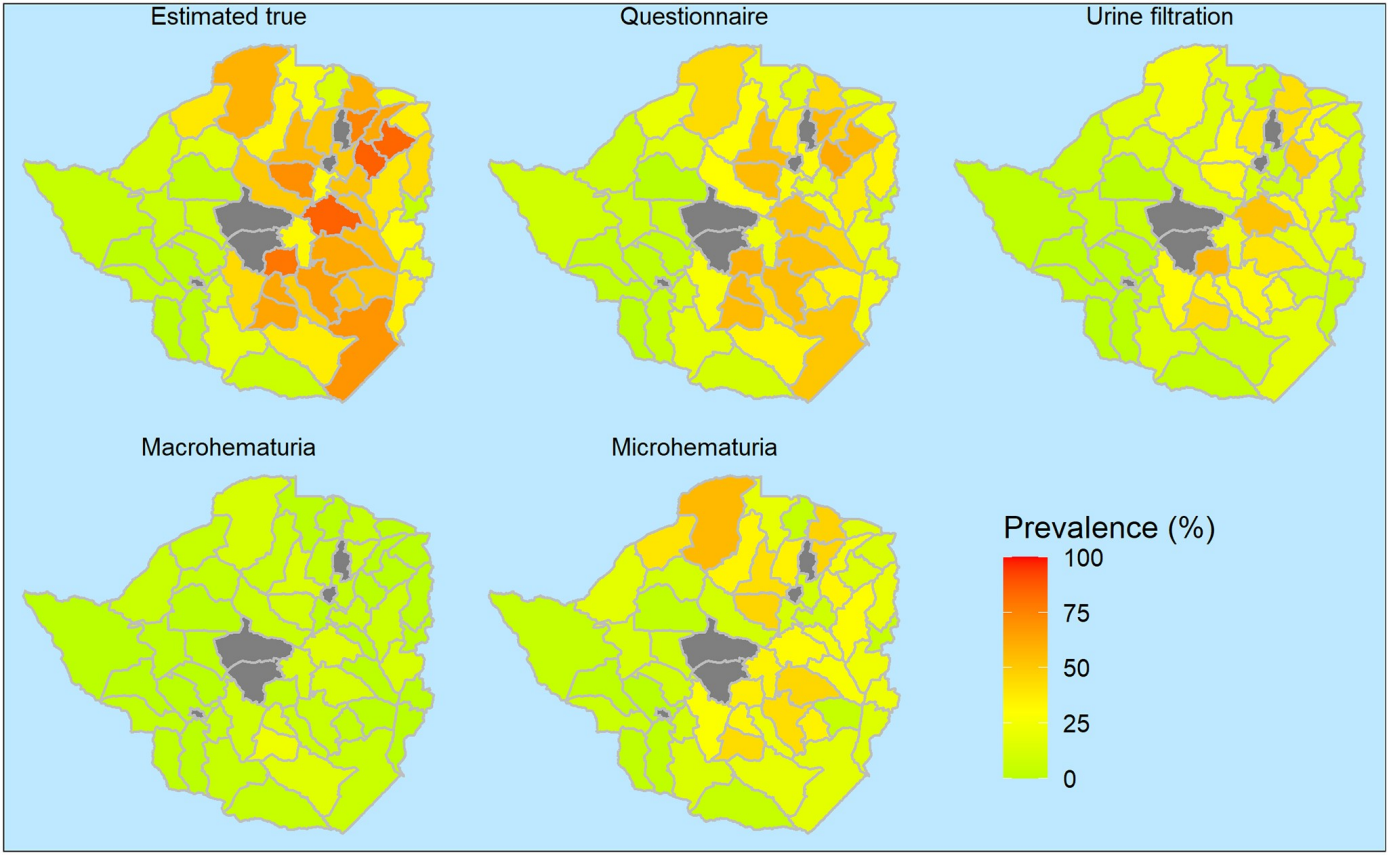
Estimated ‘true’ and observed prevalence of *S*. *haematobium* in Zimbabwe by district and diagnostic method. Grey areas indicate the urban districts of Harare, Bulawayo, and the three districts where no data were collected (Zimbabwe, 2010/2011).

The urban districts of Bulawayo have much lower prevalence than Harare and Chitungwiza but there is considerable variability within a city, e.g. from 10.4% to 25.3% in Harare, and from 2.8% to 14.8% in Bulawayo (S1 Table). The estimated ‘true’ prevalence and the observed prevalence by district for the whole country are shown in Fig 1. All maps depict low prevalence in the western districts, while there is some disagreement between hematuria-based techniques showing higher prevalence in the northern districts compared to urine filtration.

### Infection intensity-dependent sensitivity

**Fig 2:**
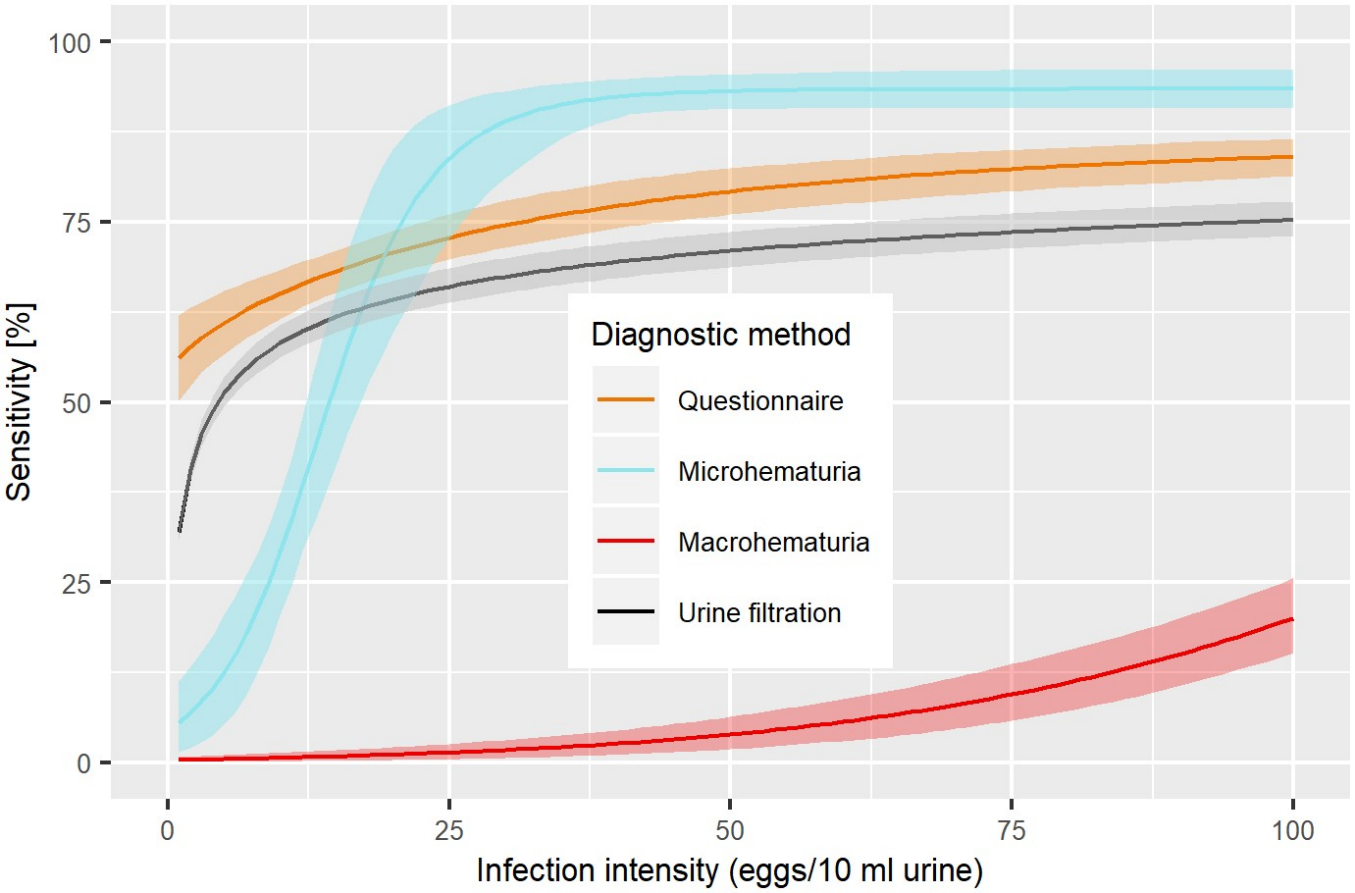
Infection intensity-dependent sensitivity estimates for urine filtration, microhematuria, macrohematuria, and a questionnaire for diagnosis of *S*. *haematobium*(Zimbabwe, 2010/2011).

All four diagnostic methods show a clear dependence of sensitivity on infection intensity, as determined by the urine filtration (Fig 2). Urine filtration, microhematuria, and the questionnaire reached considerable sensitivity (> 60%) at an intensity of 25 eggs/10 ml of urine. While the sensitivity of the questionnaire did not surpass 90%, microhematuria detected almost all cases with an intensity of more than 50 eggs/10 ml of urine. The sensitivity of macrohematuria stayed very low up to moderate infection intensities with only 25% at 100 eggs/10 ml of urine and less than 10% at 50 eggs/10 ml of urine. The questionnaire shows the weakest dependence on infection intensity with sensitivity above 50% even at very low infection intensities.

### Evaluation of treatment needs

The WHO defines treatment needs according to prevalence thresholds at 10% and 50% based on parasitologic methods. Table 3 presents estimates of the corresponding thresholds of hematuria-based diagnostics. A 10% prevalence observed by urine filtration corresponds to a ‘true’ prevalence of 18.5% and an observed prevalence of 17.9%, 13.3%, and 2.7% based on the questionnaire, microhematuria, and macrohematuria, respectively.

**Table 3:** Prevalence thresholds of hematuria-based diagnostic methods corresponding to urine filtration ones estimated from a national survey conducted in Zimbabwe in 2010/2011.

	Threshold 1 (%)	Threshold 2 (%)
Urine filtration	10	50
True prevalence	18.5	92.9
Questionnaire	17.9	62.7
Microhematuria	13.3	48.8
Macrohematuria	2.7	10.7

Only two districts had urine filtration-based prevalence of *S*. *haematobium* above 50%. Hence, we show the district classification results for the treatment category of 10-49%. There were 40 districts with *S*. *haematobium* prevalence above 10% and 30 districts with prevalence below this threshold, giving a decent sample size for both groups. Table 4 compares the ability of each diagnostic method to classify a district in the correct treatment category. All three methods showed few false positives, i.e., 4, 4, and 7 for the questionnaire, microhematuria and macrohematuria, respectively. The questionnaire and microhematuria classified 35 and 34, or 87.5% and 85.0%, respectively, of the 40 districts in the correct treatment category, while macrohematuria does so only for 62.5% of the districts (S2 Table).

**Table 4:** Ability of each diagnostic method of detecting districts with treatment needs according to WHO (10-49% parasitologic) using the adjusted thresholds.

	Urine filtration > 10%, n = 40	Urine filtration < 10%, n = 30
Questionnaire > 17.9%	35	4
Questionnaire < 17.9%	5	26
Microhematuria > 13.3%	34	4
Microhematuria < 13.3%	6	26
Macrohematuria > 2.7%	25	7
Macrohematuria < 2.7%	15	23
Questionnaire > 10%	38	11
Questionnaire < 10%	2	19
Microhematuria > 10%	38	9
Microhematuria < 10%	2	21
Macrohematuria > 10%	8	1
Macrohematuria < 10%	32	29

**Fig 3:**
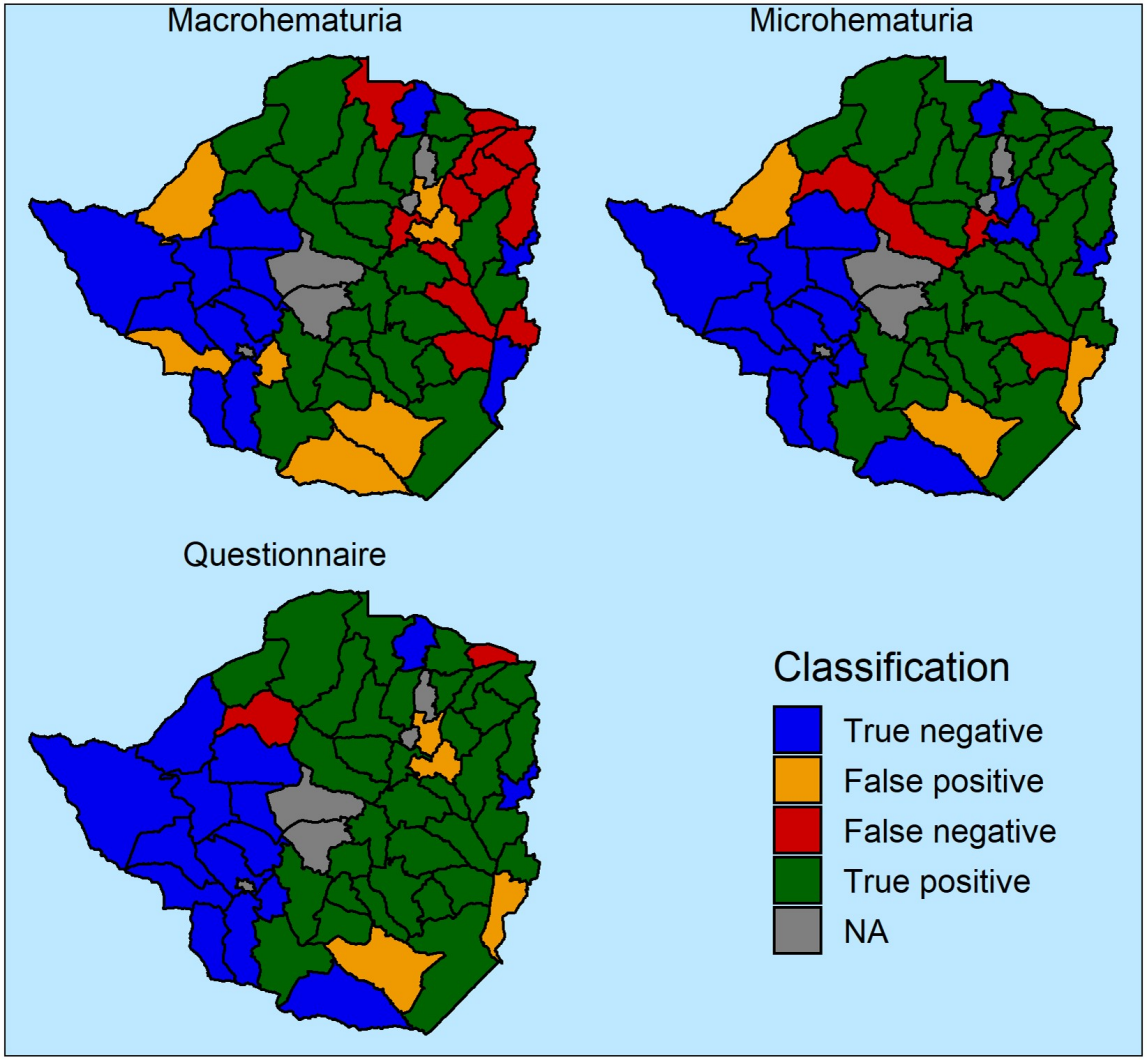
Classification of districts according to hematuria-based diagnostic methods. True negative indicates classification below threshold for both urine filtration and the given alternative; false positive indicates exceeding threshold in alternative but not in urine filtration; false negative indicates urine filtration above threshold but alternative below; and true positive indicates both urine filtration and alternative above threshold.

Sensitivity and specificity can be represented in a receiver operating characteristics (ROC) curve when the threshold is varied from 0% to 100% (S1 Fig). Both microhematuria and the questionnaire show very similar curve with sensitivity of 95% reached at specificities between 70% and 80%, and specificity of 95% at sensitivities between 55% and 65%. Macrohematuria shows lower accuracy with 95% specificity at 35% sensitivity and 70% sensitivity at 60% specificity. The classification of districts to a treatment category (Fig 3) indicates that there was no spatial pattern in the districts that were wrongly excluded from treatment for microhematuria and the questionnaire, while macrohematuria missed districts that needed treatment mostly in the north-western part of Zimbabwe.

**Fig 4:**
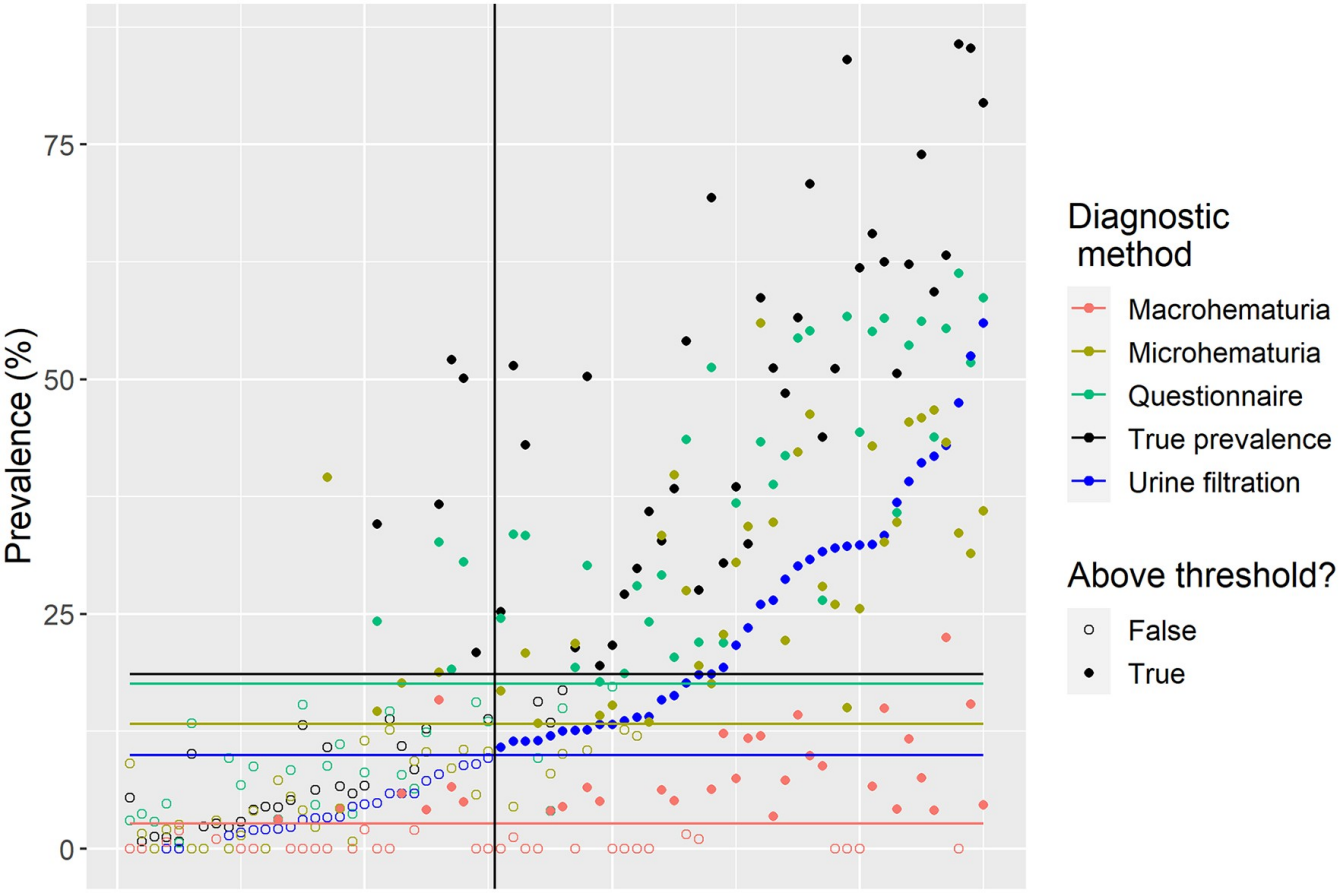
District prevalence for all 70 districts surveyed in Zimbabwe and the four diagnostic methods including the estimated ‘true’ prevalence ordered by urine filtration prevalence on the x-axis. The vertical line indicates the 10% urine filtration prevalence border. Filled dots to the left of this line are false positive, while empty dots to the right are false negatives. The horizontal lines show the determined threshold for each diagnostic method.

Fig 4 depicts the district prevalence measured by each diagnostic method, ordered by urine filtration. False positives by questionnaire were all in districts with urine filtration above 5%. The same is true for microhematuria with one outlier recording 40% observed prevalence in a district with < 5% positives according to urine filtration. Macrohematuria determined treatment needs in districts with < 5% urine filtration and simultaneously missed districts approaching 50%. The districts that were falsely identified as negative by microhematuria and the questionnaire were always at urine filtration prevalence below 15%.

## Discussion

This is the first study to estimate the diagnostic error of urine filtration, a parasitologic method which detects eggs in urine and of three hematuria-based methods for *S*. *haematobium* diagnosis, i.e. a questionnaire regarding recent history of blood in urine, a microhematuria reagent strip, and visual inspection of urine to detect macrohematuria. Our modeling approach took into account the dependence of sensitivity on the egg intensity. We further evaluated the ability of the three hematuria-based diagnostic techniques to predict treatment needs. Analysis was done using an adaptation of an egg count model previously utilized to determine the dependence of the Kato-Katz diagnostic technique for *S*. *mansoni* on the infection intensity and to translate intervention thresholds from Kato-Katz to the point-of-care circulating cathodic antigen (POC-CCA) urine cassette test [17, 26]. The model was fitted on a large data set stemming from a national survey in Zimbabwe with more than 15,000 school children examined for *S*. *haematobium* using a suite of diagnostic tests.

Generally, the strong dependence on infection intensity of the different diagnostic methods indicates that an assumption of constant sensitivity is not valid. An advantage of urine filtration is that its sensitivity increases by repeated sampling, which is not possible for questionnaires, but clearly also for reagent strip tests for microhematuria and inspection of urine sample for visible blood. However, this improvement cannot be measured in this study due to lack of repeats allowing the estimation of within person variability.

The questionnaire showed the highest overall sensitivity (70.0%), but it had a rather low specificity of 93.7% for individual diagnosis. Low specificity is less of an issue when adverse events of possible over-treatment are negligible. Macrohematuria shows very high specificity (> 99%), but a low sensitivity of 11.5% at the individual level. Microhematuria, with a sensitivity of 52.2% and specificity of 95.6% does not improve the diagnostic error of urine filtration. Furthermore, the effort and cost required to perform reagent strip testing for *S*. *haematobium* diagnostic is comparable to standard urine filtration.

Mafe et al. (1997) evaluated macrohematuria, microhematuria, and a different form of the questionnaire asking for the current passing of blood in urine in a study of 1,056 individuals in the Kainji Lake area of Nigeria [29]. Urine filtration was used as a reference standard, leading to overestimation of the sensitivity in a highly endemic area with no previous treatment. The level of endimicity might explain that both macrohematuria and microhematuria showed higher sensitivity (69% and 38% respectively), compared to the estimates from our study in Zimbabwe. Sensitivity of the questionnaire was low (44%), which is not surprising given that the question was about the current blood passing instead of the recent history like in our study. Fatiregun et al. (2005) compared different variants of questionnaires and a reagent strip to detect microhematuria with urine filtration as reference standard [30]. ‘Unqualified hematuria’ the most similar version of the questionnaire to this study, showed a specificity of 93.1% similar to our estimate, while the sensitivity was much lower at 41.7%. Lower sensitivity can be explained by lower overall prevalence and infection-intensity in the study setting. Koukounari et al. (2009) used latent class modeling to compensate for the lack of reference test, therefore comparison of the results is difficult [31].

Krauth et al. (2015) showed that in low prevalence settings, microhematuria is an unstable proxy for *S*. *haematobium* infections [32]. Hematuria can be caused by a number of other conditions (e.g. sickle cell disease) that have important spatial variations. We estimated specificity of the tests in girls in the age between 12 and 14 years separately but no difference was found. Our analysis could not control for additional conditions that might cause hematuria because such data were not collected. We assumed that the specificity of the diagnostic tests is constant, which may not necessarily be true when other conditions, possibly spatially varying, can cause false-positives. The absence of a well performing diagnostic test for *S*. *haematobium* motivates the development of alternative diagnostics based on either specific antigens, or molecular markers, but none of them is currently accurate and cost-effective enough to use in large scale surveys [12, 33–36].

Our results do not mean that hematuria-based diagnostic techniques cannot be used to evaluate treatment needs. Lengeler et al. (2000) showed that questionnaires perform well in identifying schools at high-risk of *S*. *haematobium* in a large study in the Democratic Republic of the Congo and a review of studies from 10 countries, involving a total of 133,880 children [10, 37]. These studies revealed a striking relation between the prevalence observed by parasitologic methods and the number of positive responses to the questionnaire. Sensitivity at the community level was in the order of 90% for most countries, while specificity was mostly above 80% with the exception of Cameroon, Malawi and Zimbabwe.

In the current national survey of Zimbabwe using an intervention prevalence threshold of 17.9% for the questionnaire (equivalent to 10% urine filtration) almost 90% of districts with treatment needs were correctly identified, while the specificity was similarly high (> 85%). Misspecified districts showed urine filtration prevalence between 5% and 15% representing a range of prevalence where differences in treatment decisions can be justified. Thus, the data support that a simple questionnaire can be used as a low effort diagnostic alternative to collecting urine for filtration for evaluating treatment needs. The suggested threshold of 17.9% has to be estimated prospectively in different settings at different stages of control. Microhematuria performs equally well to the questionnaire for predicting treatment needs but requires the collection of urine samples. Hence, the advantage of the method compared to urine filtration is reduced. Macrohematuria shows a 62.5% sensitivity for district-level treatment needs and a lower specificity of 76.7%. Misclassified districts by macrohematuria cover almost the whole range of urine filtration prevalence from < 5% to almost 50%.

### Conclusion

We showed that a simple questionnaire has high diagnostic accuracy in predicting treatment needs for *S*. *haematobium* at a level of 10% urine filtration when a threshold of 17.9% is used. The threshold suggested here has to be evaluated and confirmed in other settings. The corresponding specificity was high in Zimbabwe but this is not necessarily the case for other locations due to varying prevalence of hematuria causing conditions. Still, the low effort of performing questionnaire surveys compared to urine filtration can make the questionnaire a promising tool for low-cost rapid assessment. For optimal individual diagnosis, urine filtration is still the best method among the ones tested in this study due to its high specificity, the possibility of increasing its sensitivity through repeated testing, and the additional information that is provided regarding the infection intensity.

## Supporting information

S1 Fig Diagnostic error of district classification in treatment categories (receiver operating characteristics (ROC)) based on prevalence thresholds of the questionnaire, microhematuria and macrohematuria corresponding to a 10% urine filtration prevalence. (TIF)

S1 Table Observed and ‘true’ estimated prevalence of urban districts in Harare, Bulawayo, and Chitungwiza of Zimbabwe in a national survey conducted in 2010/2011. (PDF)

S2 Table Sensitivity and specificity of three hematuria-based diagnostic method for determining the treatment need in a national survey in Zimbabwe conducted in 2010/2011. (PDF)
