# Supplementary material for: Accuracy of different diagnostic techniques for Schistosoma haematobium to estimate treatment needs in Zimbabwe: Application of a hierarchical Bayesian egg count model
Source: PLoS Negl Trop Dis. 2020 Aug 20;14(8):e0008451. doi: 10.1371/journal.pntd.0008451 (PMC7462259; doi:10.1371/journal.pntd.0008451)
Supplement: S1 Table — (PDF) [file pntd.0008451.s002.pdf]

S1: Observed and 'true' estimated prevalence of urban districts in Harare, Bulawayo, and Citungwiza of Zimbabwe in a national survey conducted in 2010/2011

(%)	True prevalence	Questionnaire	Urine filtration	Macrohematuria	Microhematuria
**Harare**					
Peri-urban	25.3 (15.8 - 36.3)	24.6	10.8	0.0	16.8
Glen View / Mufakose	26.3 (18.9 - 36.2)	18.7	13.6	0.0	12.6
Highfields / Glen Norah	13.6 (8.8 - 19.8)	13.5	9.7	0.0	10.3
Mabelreign / W-Park	16.3 (10.6 - 22.7)	9.7	11.5	0.0	13.4
Mabvuku / Tafara	13.2 (1.7 - 26.0)	8.8	3.3	0.0	39.6
Mbare / Hatfield	15.9 (7.4 - 21.7)	14.6	5.9	0.0	12.7
Greystone Park	10.4 (0.3 - 35.4)	13.0	0.0	0.0	0.0
**Bulawayo**					
Peri-urban	2.8 (1.1 - 6.1)	6.8	1.7	0.0	1.4
Rogate	5.1 (2.5 - 12.1)	4.7	3.3	0.0	2.3
Imbizo	2.2 (0.0 - 8.6)	0.0	0.0	0.0	0.0
Khami	5.9 (2.4 - 11.5)	3.7	4.4	0.0	0.7
Mzilikazi	4.4 (0.6 - 12.1)	0.0	2.0	0.0	0.0
Sizinda	14.8 (4.8 - 25.8)	4.0	12.0	4.0	8.0
**Chitungwiza**					
Seke-Chitungwiza	20.4 (10.8 - 31.9)	15.6	9.0	0.0	5.7
Zengeza	2.6 (0.0 - 9.4)	9.6	1.4	0.0	0.0
