# Supplementary material for: Accuracy of different diagnostic techniques for Schistosoma haematobium to estimate treatment needs in Zimbabwe: Application of a hierarchical Bayesian egg count model
Source: PLoS Negl Trop Dis. 2020 Aug 20;14(8):e0008451. doi: 10.1371/journal.pntd.0008451 (PMC7462259; doi:10.1371/journal.pntd.0008451)
Supplement: S2 Table — (PDF) [file pntd.0008451.s003.pdf]

S2: Sensitivity and specificity of three hematuria-based diagnostic method for determining the treatment need in a national survey in Zimbabwe conducted in 2010/2011.

	District sensitivity	District specificity
Questionnaire 17.9%	87.5%	86.7%
Microhematuria 12.4%	85.0%	86.7%
Macrohematuria 2.6%	62.5%	76.7%
Questionnaire 10%	95.0%	63.3%
Microhematuria 10%	95.0%	70.0%
Macrohematuria 10%	20.0%	96.7%
